# Interactive Block Games for Assessing Children's Cognitive Skills: Design and Preliminary Evaluation

**DOI:** 10.3389/fped.2018.00111

**Published:** 2018-05-08

**Authors:** Kiju Lee, Donghwa Jeong, Rachael C. Schindler, Laura E. Hlavaty, Susan I. Gross, Elizabeth J. Short

**Affiliations:** ^1^Distributed Intelligence and Robotics Laboratory, Department of Mechanical and Aerospace Engineering, Case Western Reserve University, Cleveland, OH, United States; ^2^Department of Pediatrics (Secondary), Case Western Reserve University, Cleveland, OH, United States; ^3^Department of Psychological Sciences, Case Western Reserve University, Cleveland, OH, United States

**Keywords:** block games, cognitive assessment, block design test, technology-based assessment, child development

## Abstract

**Background:** This paper presents design and results from preliminary evaluation of Tangible Geometric Games (TAG-Games) for cognitive assessment in young children. The TAG-Games technology employs a set of sensor-integrated cube blocks, called SIG-Blocks, and graphical user interfaces for test administration and real-time performance monitoring. TAG-Games were administered to children from 4 to 8 years of age for evaluating preliminary efficacy of this new technology-based approach.

**Methods:** Five different sets of SIG-Blocks comprised of geometric shapes, segmented human faces, segmented animal faces, emoticons, and colors, were used for three types of TAG-Games, including Assembly, Shape Matching, and Sequence Memory. Computational task difficulty measures were defined for each game and used to generate items with varying difficulty. For preliminary evaluation, TAG-Games were tested on 40 children. To explore the clinical utility of the information assessed by TAG-Games, three subtests of the age-appropriate Wechsler tests (i.e., Block Design, Matrix Reasoning, and Picture Concept) were also administered.

**Results:** Internal consistency of TAG-Games was evaluated by the split-half reliability test. Weak to moderate correlations between Assembly and Block Design, Shape Matching and Matrix Reasoning, and Sequence Memory and Picture Concept were found. The computational measure of task complexity for each TAG-Game showed a significant correlation with participants' performance. In addition, age-correlations on TAG-Game scores were found, implying its potential use for assessing children's cognitive skills autonomously.

## 1. Introduction

The development of cognitive skills begins at birth and continues throughout adulthood (1, 2). Optimizing development and identifying cognitive issues that place children at risk for developmental delays has been the mission of pediatricians, psychologists, and educators alike (3, 4). More recently, politicians have joined the movement mandating the use of standardized cognitive assessment tests at frequent intervals throughout a student's academic career. A plethora of tests are available to be used for periodic assessment and their reliability and validity are well established. However, the standardized nature often requires an unvarying set of problems, presented in a prescribed sequence, and a trained specialist to administer the test. Among the earliest and most frequently used assessment instruments, the Wechsler series (5, 6) is considered to be the “gold standard” (7). The Wechsler series has two age standardized variants, exclusively designed for children: the Wechsler Preschool and Primary Scale of Intelligence – 4th Edition (WPPSI-IV) for ages 2:6–7:7 and Wechsler Intelligence Scale for Children-5th Edition (WISC-V) for ages 6:0–16:11. To minimize language bias, Raven's Progressive Matrices (RPM) are sometimes used in place of the Wechsler tests (8, 9). RPM involves a series of perceptual analytic reasoning problems presented in a matrix format, which do not rely on language usage and thus appear to reduce cultural bias (10, 11). Pediatricians and psychologists often use these standardized tests in conjunction with parental interviews and clinical observations to pinpoint a child's strengths and weaknesses and thereby develop an appropriate developmental/educational intervention plan (12, 13).

While cognition, attention, memory, and language are strong components of traditional intellectual assessments, motor skills have played less of a role. Nonetheless, motor control and hand-eye coordination skills are found to be closely linked with child development as well as general cognitive and learning abilities (14–17). Only a few instruments target these skills directly. The two such examples are Block Design (BD) in the Wechsler test series and the Beery Visual-Motor Integration (VMI) test. BD uses a set of red-and-white blocks where the examinee must copy an abstract image by assembling these blocks. This test, designed to measure spatial visualization and motor skills, is considered the best single predictor of Performance IQ (18–20). VMI measures both cognitive processing and motor response by asking the individual to trace geometric drawings using a pencil (21).

The reliability and validity of standardized measures, such as the Wechlser series, RPM, and the VMI, are well established; however, the challenges associated with obtaining accurate, objective, and timely assessment of cognitive skills are still formidable. The most pressing challenges include the costs associated with testing (22), limited availability of appropriately trained clinicians (23), and difficulties in addressing individual differences in age and cognitive status (24). Cognitive tests require trained professionals to administer and manually record the performance of the examinees in terms of speed and accuracy. Aside from high operation costs, this process is also susceptible to human errors. For children, administration of standardized tests become more challenging because of language demands, attentional fluctuations, and lack of comfort (25).

In an attempt to address the above challenges, researchers have been investigating computerized approaches for cognitive assessment in children. Many traditional forms of paper-pencil tests have been converted into computerized forms. For example, Pearson's Q-interactive allows iPad-mediated administration of cognitive tests, including WISC-V and WPPSI-IV (26). While Q-interactive reduces clinician's workload and increases engagement in examinees, the level of automation is still limited and requires a professional for administration (26, 27). Computerized psychomotor tests, including finger tapping test, simple reaction time, choice reaction time, choice discrimination test, digit picture substitution test, and card sorting test, were employed for cognitive assessment in children with learning disabilities (28). A recent review reported that gamified cognitive tests, using a computer or other technical tools, were highly engaging and reduced anxiety and thus improved motivation (29). Despite the potential, most of computer-based methods have been focused on automating scoring, rather than automating administration (30). In addition, there is no clinically valid computerized tool that can fully automate tasks, involving physical object manipulation, such as the Wechsler's BD subtest. There exist some studies on technology-assisted approaches, but the technical functionality in these works was quite primitive, such as a tabletop interface with a stereo camera providing limited assessment data on the user performance (31).

**Figure 1:**
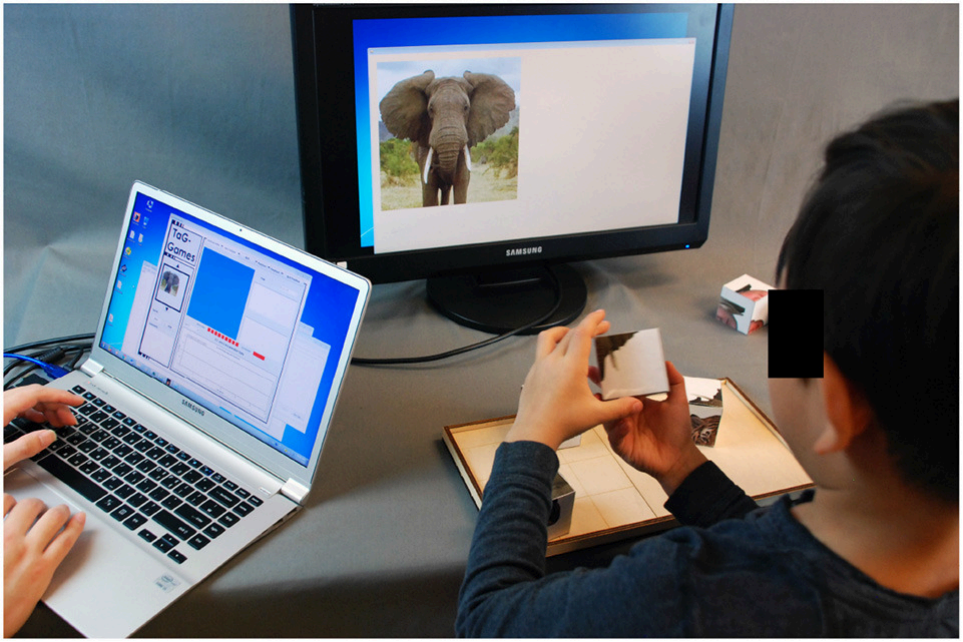
Study setting: The TAG-Games technology consists of a set of SIG-Blocks, an interfacing computing device (e.g., a laptop) for administration and monitoring, and an extra screen for displaying test items. This picture shows a child using four SIG-Blocks with segmented animal faces to match the displayed image. Parent consent was obtained for publication of this image.

In this paper, a technology-based approach using sensor-integrated geometric blocks (SIG-Blocks) was employed for cognitive assessment in young children (See Figure 1) (32–34). The computerized tests using SIG-Blocks are called TAG-Games. Building on our previous work involving 98 university students (32), this technology-based instrument has been redesigned for the target age group and tested on 40 participants at the ages of 4–8. The system employs a set of SIG-Blocks and an interfacing computing device, such as a laptop computer with two screens (or two computers) for TAG-Game administration. One screen is used for real-time administration and monitoring of the process and the other for displaying test items to the examinee. Three sets of TAG-Games, including Assembly (TAG-Game^A^), Shape-Matching (TAG-Game^S^), and Memory (TAG-Game^M^), were developed for preliminary reliability and validity evaluation. While requiring further evaluation studies building on the preliminary, yet important, outcomes presented in this paper, this system is equipped and designed for automating both administration and scoring.

## 2. Materials and methods

### 2.1. Technology overview

TAG-Games are an integrated tangible game technology for automated test administration, visualization, and data collection (Figure 1). The games are designed to measure cognitive problem solving, working memory, and spatial reasoning skills coupled with motor responses through three sets of games, i.e., TAG-Game^A^, TAG-Game^S^, and TAG-Game^M^, using SIG-Blocks. In addition to overall accuracy and speed typically measured in existing cognitive tests, this technology-based system also assesses step-by-step procedural accuracy and speed information throughout the problem solving process. The user interface allows the administrator to monitor the examinee's real-time performance locally or remotely through a wireless network. The entire technology will be available at a relatively low cost, with the estimated commercial price of the TAG-Games package to be less than $1,000. This paper presents design and preliminary evaluation of TAG-Games for cognitive assessment in young children. TAG-Games were previously examined for the technical functionality and preliminary utility in assessing cognitive skills of adults. In keeping the overall design of TAG-Games similar to the adult version, we modified the TAG-Games tests carefully aiming to be appropriate for the target age group of 4–8. Specifically, the following modifications were made: (1) SIG-Blocks covered with segmented human and animal faces and simple emoticons were added and used in the new TAG-Games; (2) Easier items were added and harder ones were removed; and (3) Discontinuation rule was applied by stopping the test after the child fails to answer correctly on two consecutive items at the same difficulty level. More details about the hardware, game design, and computational complexity measures are followed.

#### 2.1.1. Hardware design of SIG-blocks (32, 34)

**Figure 2:**
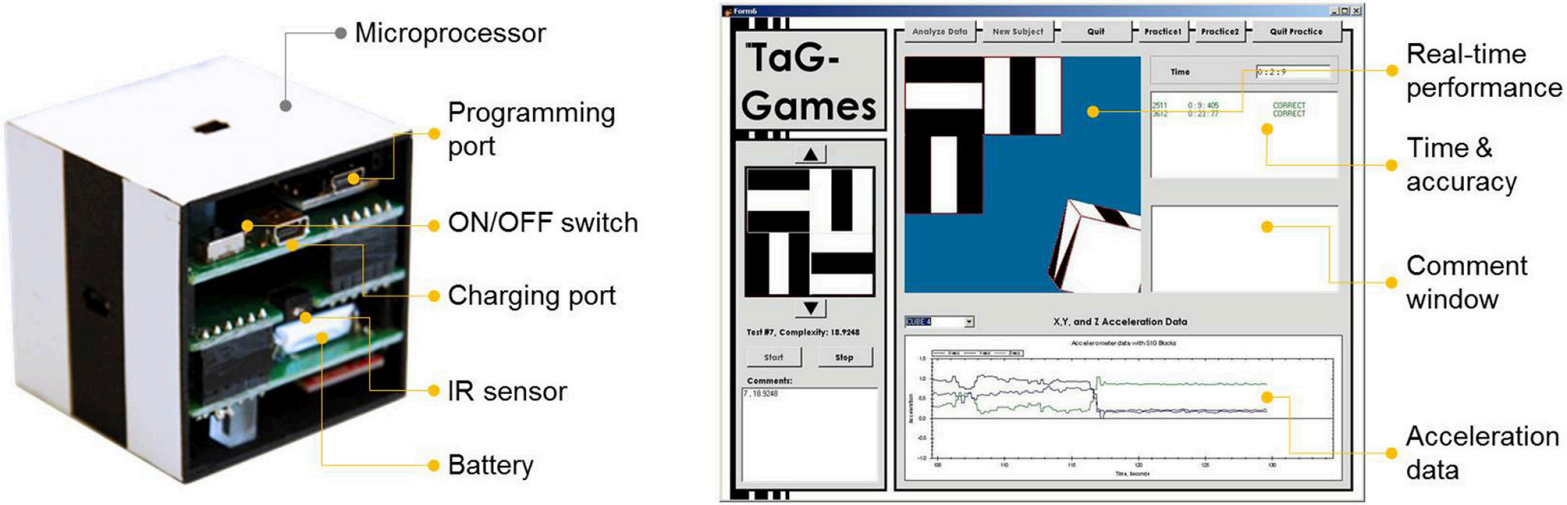
Hardware design and embedded electronic components of SIG-Block and GUI layout for administrator, displaying the item, current assembly configuration, correctness with time stamp at each manipulation step, and real-time accelerations of the block (32).

Embedded in the system are six optical sensors, a tri-axial accelerometer, a ZigBee-based wireless communication module, and a timer in the microprocessor, which are used to determine the accuracy and time for each manipulation step and wirelessly transfer to an interfacing device (Figure 2). Algorithms were developed to measure accuracy and speed at each manipulation step. A single step of manipulation refers to when any two blocks were assembled together. For example, if an item required the person to assemble four blocks to achieve a specific assembly configuration, the minimum number of manipulation steps is three; however, the person could make more than three manipulations. The system records the total number of manipulation steps, the correctness, and the time for each.

#### 2.1.2. TAG-game design (32)

The target measures of TAG-Games are fine motor, visual-motor integration, problem solving and working memory skills. A unique feature of the presented system is the use of physical objects, i.e., SIG-Blocks. In order to capture target cognitive skills, three types of TAG-Games (i.e., TAG-Game^A^, TAG-Game^S^, and TAG-Game^M^) were designed to selectively measure subsets of these domains. Table 1 shows three types of TAG-Games and the cognitive skills which are expected to be associated with each game. TAG-Game^A^ is an assembly construction game in which the user recreates a displayed image by assembling the SIG-Blocks. The displayed item is an arrangement of the images on the block faces. The player must rotate and rearrange the blocks in order to create the displayed pattern. TAG-Games^S^ involves the presentation of assembly patterns with a missing piece displayed. The participant is prompted to fill in the missing image by placing a SIG-Block with the matching image. For all items, the system records the time it takes for the participant to complete the pattern and whether it is completed correctly or not. As with TAG-Game^A^, TAG-Game^S^ requires fine-motor control, as well as visuospatial reasoning (i.e., ability to see the relationships between block rotations and face images) and problem-solving skills (i.e., finding the relations within the pattern and predicting the missing image). TAG-Game^M^ requires the participant to remember a sequence of images and repeat it back using a SIG-Block. The images within the sequence are flashed one at a time on a screen, and the participant repeats the sequence by placing the SIG-Blocks with the correct image face up in the order that they appeared. TAG-Game^M^ is designed to test a participant's fine motor control, working memory, and attention span. Fine motor control is reflected in the speed and accuracy with which the participant can rotate the block to find the right face image. Working memory is reflected in how well the participant remembers the sequence of images. Attention span is reflected in the ability to maintain focus when the sequences become longer.

**Table 1:** Three types of TAG-Games and cognitive skills expected to be associated with each game.

**Type**	**Associated cognitive and motor skills**
TAG-Game^A^	Fine-motor proficiency
	Visual-motor integration
	Low-level working memory
TAG-Game^S^	Fine-motor proficiency
	Visual-motor integration
	Low-level working memory
	Spatial reasoning
TAG-Game^M^	Fine-motor proficiency
	Visual-motor integration
	High-level working memory

#### 2.1.3. Computational complexity measures (32)

Computational measures of play complexity (*C*^*play*^) were defined for each TAG-Game. *C*^*play*^ measures relative complexity of the items used in each game, based on the number of blocks and their geometric properties (including rotational symmetry and color). We hypothesized that these complexity measures were correlated to the performance and by adjusting these factors affecting *C*^*play*^, the administrator could fine-tune the test difficulty for a target population or individual. For TAG-Game^A^, $C_{A}^{play}$ was defined as a configurational entropy change during an assembly task. The change was found from the difference between the entropy before (*H*^*initial*^) and after (*H*^*final*^) the task. For TAG-Game^S^, $C_{S}^{play}$ increased as the number of blocks (*N*), the number of distinctive images used in the item (*N*_*d*_), and/or the pattern length (*L*) increased and decreased as the number of pattern repeats (*R*) and/or the number of symmetry axes (*S*) increased. For TAG-Game^M^, $C_{M}^{play}$ was calculated by counting the total number of possible arrangements for the images used in the item (*Q*) and the length of the sequence (*L*) and then taking the base-2 logarithm. Specific formulas used for each TAG-Game are provided below:

$$\begin{array}{rcl} C_{A}^{play} & = & H^{initial}-H^{final};C_{S}^{play}=\frac{N\cdot N_{d}\cdot L}{R\left(S+1\right)}; \end{array}$$

(1) $$\begin{array}{rcl} C_{M}^{play} & = & \log_{2}Q+\log_{2}L \end{array}$$

**Figure 3:**
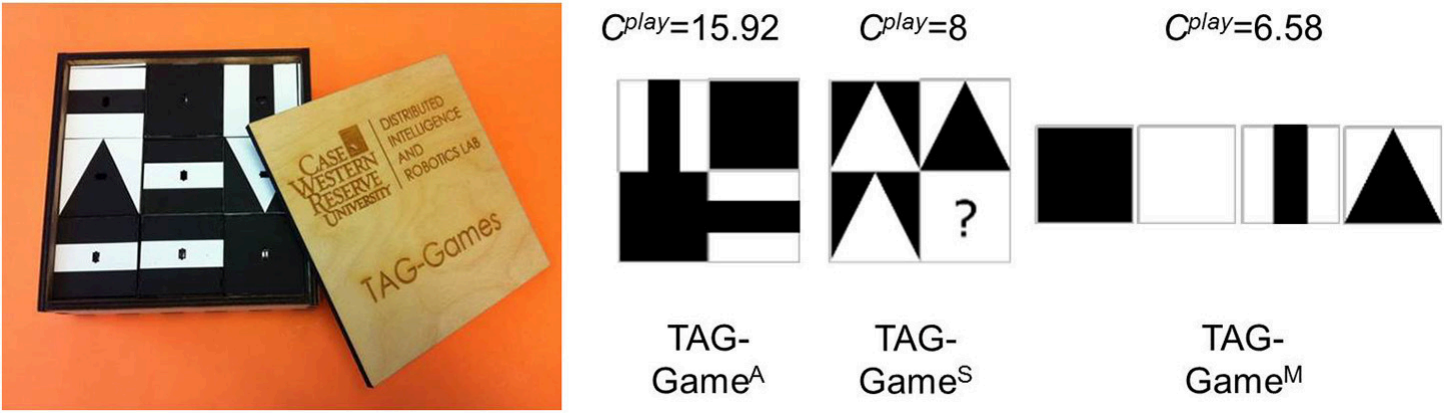
SIG-Block cover images: For children's TAG-Games, in addition to the geometric shapes and color blocks used in our preliminary study, SIG-Blocks with segmented human and animal face images and emoticons were also used. *C*^*play*^ values are calculated by the formula shown in Equation (1).

Figure 3 shows a set of SIG-Blocks and example TAG-Game items and their corresponding *C*^*play*^ values calculated using the above formulas.

### 2.2. Study design and method

Evaluation of children's TAG-Games focused on technical functionality, validity of the proposed complexity measures that were previously verified for adults' data, and preliminary validity of the collected data in terms of assessing children's cognitive skills in comparison with a selected standardized instrument. This study was reviewed and approved by Case Western Reserve University's Institutional Review Board. Informed consent was obtained from parents of all participants. Oral assent was obtained from each child participant after a brief description of the study provided by the research team.

#### 2.2.1. Participants

Forty typically developing children (28 males), aged 4–8, participated in this study. Children were recruited for this study from an advertisement placed on the community message board at a local university in the Cleveland Area. All parents were informed of the voluntary nature of the study. Parents were thanked for their participation with a brief report about the study and children received a small educational toy of their choosing for participation.

#### 2.2.2. Protocol

**Figure 4:**
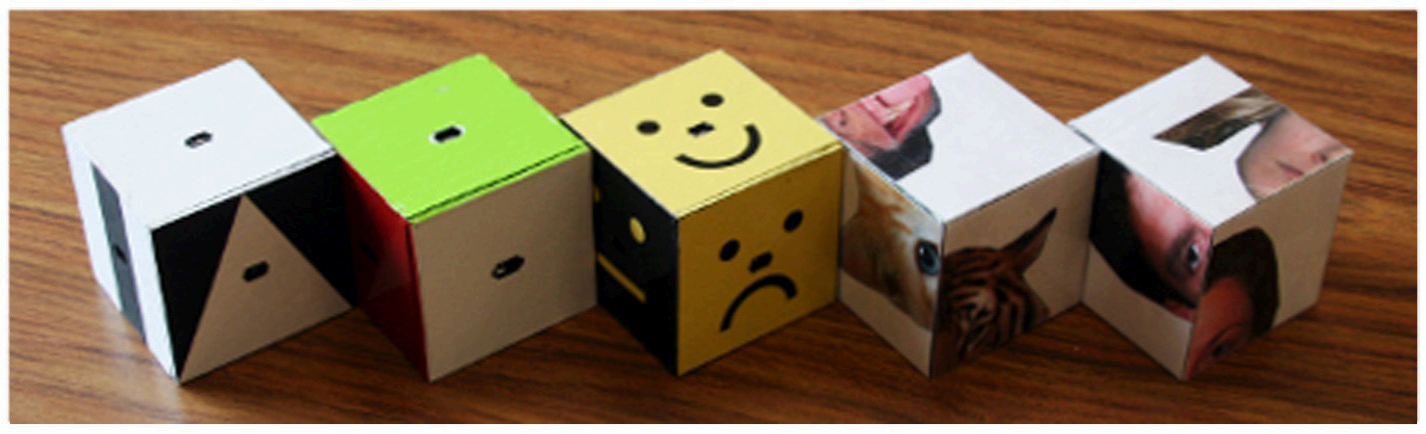
Five types of SIG-Blocks used in the study: geometric shape (GS), color (CL), emoticon (EM), segmented animal face (AF), and segmented human face (HF).

**Figure 5:**
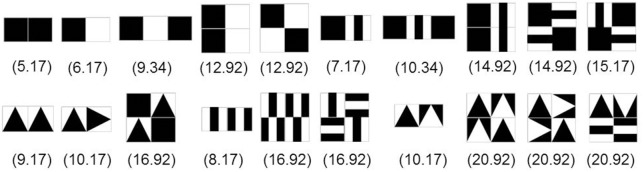
Twenty TAG-Game^A^-GS items and calculated $C_{A}^{play}$ values.

**Figure 6:**
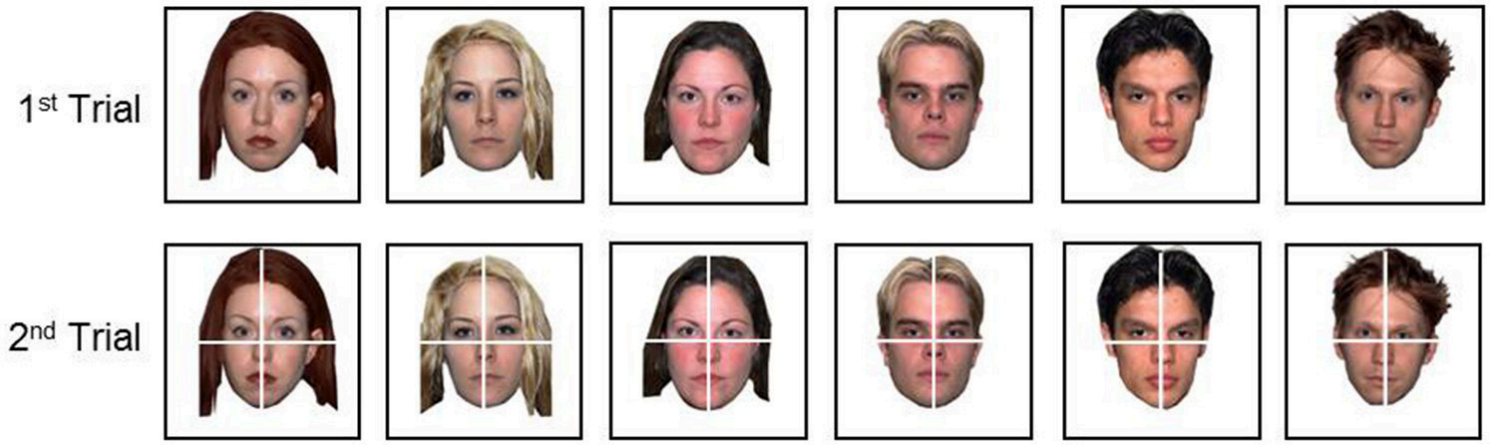
Six TAG-Game^A^-HF items. If one does not successfully assemble the blocks correctly to match the face in the first trial, the second trial shows thin grid lines to separate the image segments. $C_{A}^{play}=22.92$ for all items.

**Figure 7:**
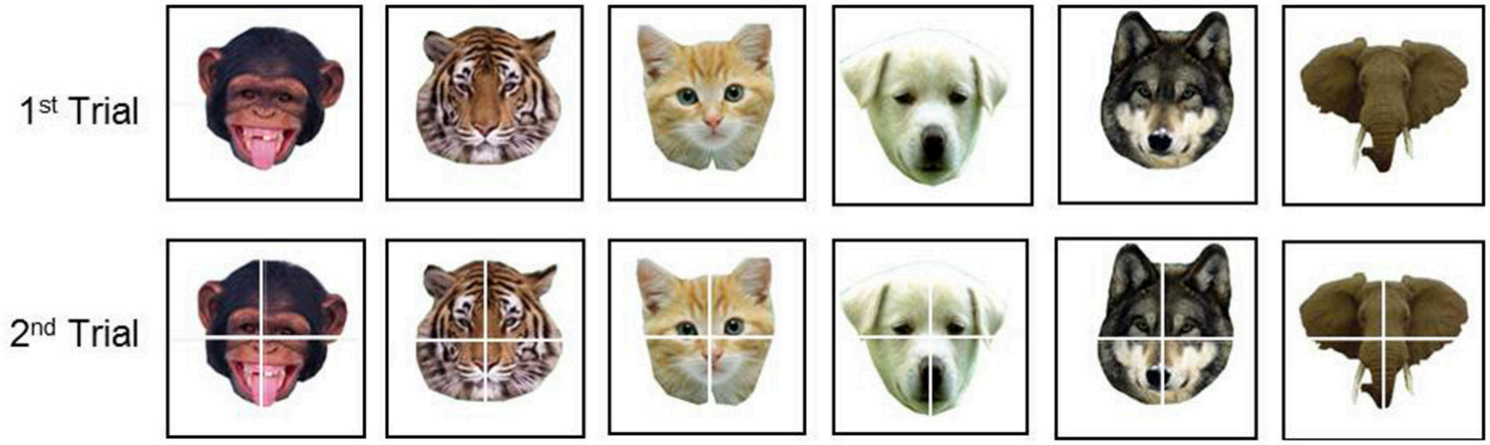
Six TAG-Game^A^-AF items. If one does not successfully assemble the blocks correctly to match the face in the first trial, the second trial shows thin grid lines to separate the image segments. $C_{A}^{play}=22.92$ for all items.

**Figure 8:**
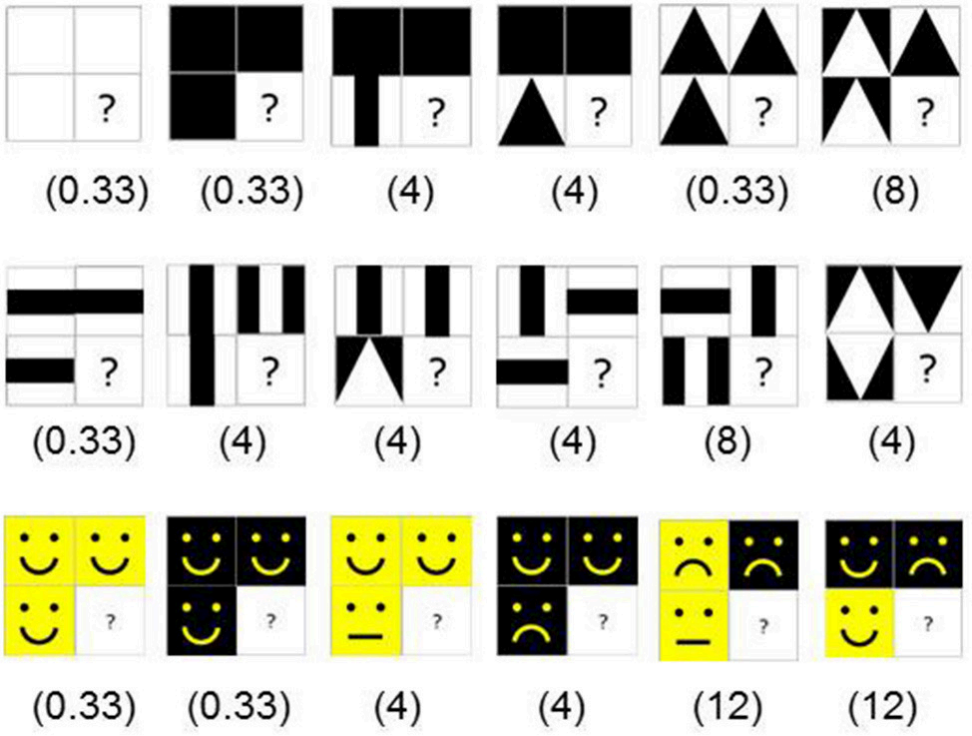
Twelve TAG-Game^M^-GS items on the first two rows and 6 TAG-Game^M^-GS items on the third row with corresponding $C_{S}^{play}$ values.

**Figure 9:**
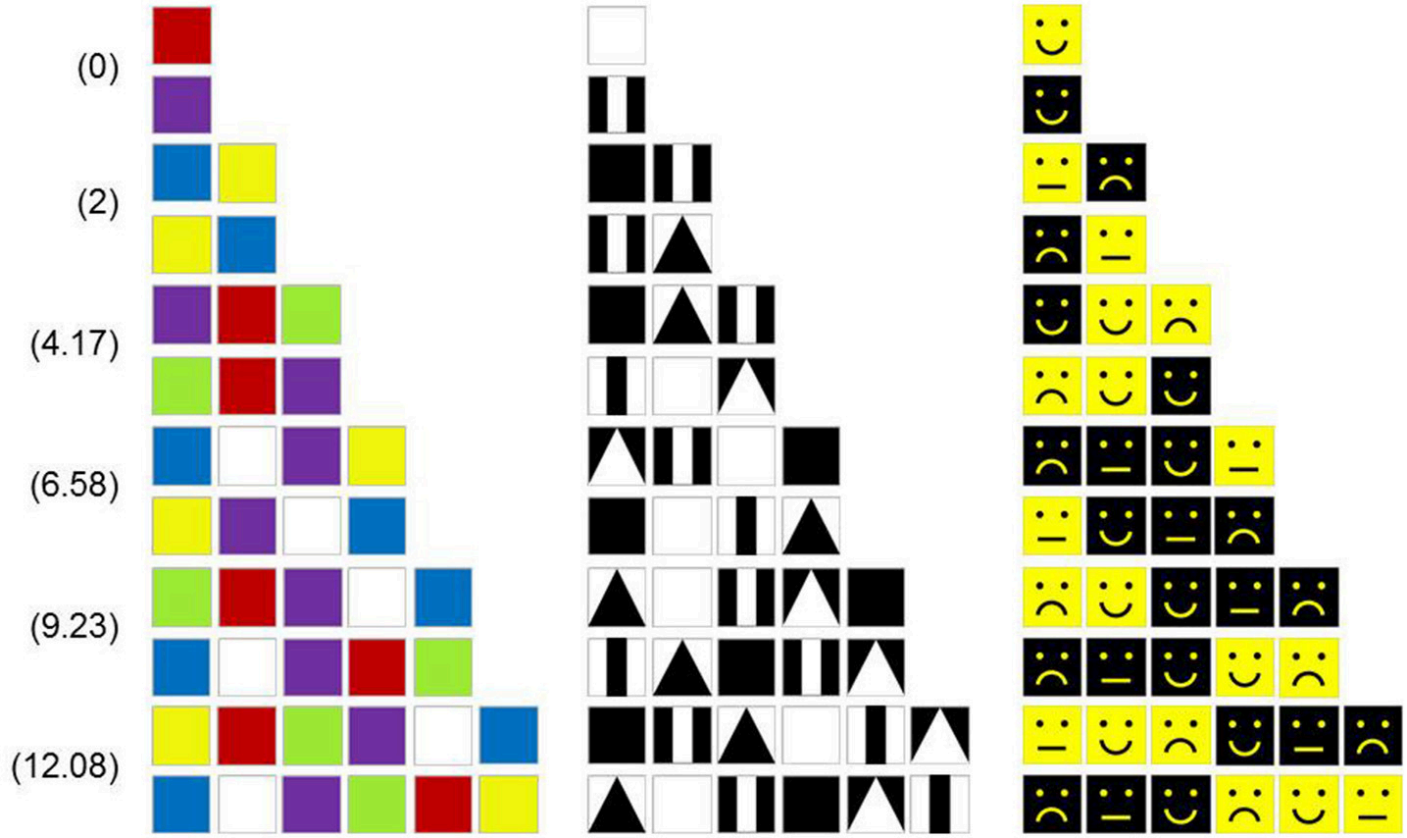
Twelve TAG-Game^M^-CL items **(Left)**, 12 TAG-Game^M^-GS items **(Middle)**, and 12 TAG-Game^M^-EM items **(Right)** with corresponding $C_{S}^{play}$ values.

TAG-Games for children employed five different designs of SIG-Blocks: geometric shapes (GS), segmented human faces (HF), segmented animal faces (AF), emoticons (EM), and colors (CL) (Figure 4). TAG-Game^A^ included three subtests, using the blocks covered with GS (20 items), HF (6 items), and AF (6 items). GS used two to four blocks while HF and AF used four blocks (Figures 5–7). For HF and AF items, if a child fails to assemble four blocks to reconstruct a displayed face image correctly, the second trial displays the same image with grid lines to separate each segment, as shown in Figures 6, 7. Two subtests were designed for TAG-Game^S^: one set of items using GS (12 items) and the other set using EM (6 items) (Figure 8). TAG-Game^M^ consisted of three subtests, each using GS, CL, or EM (Figure 9). Each subtest in TAG-Game^M^ included 6 items. A discontinuation rule was applied for TAG-Games when a participant failed to correctly answer two consecutive items. The number of items and average time for completion for each subtest are summarized in Table 2.

**Table 2:** The number of items and the average administration time for six subtests of TAG-Games.

**Test**	**Subtest**	**Max. No. Items**	**Ave. Time [min]**
TAG-Game^A^	GS	20	20
	HF	6	7
	AF	6	7
TAG-Game^S^	GS	12	10
	EM	6	5
TAG-Game^M^	GS	6	3
	CL	6	3
	EM	6	3

*A discontinuation rule was applied to all participants to stop the test when failed to correctly answer two consecutive items at the same difficulty level*.

For evaluating potential utility of TAG-Games as a cognitive assessment tool, three subtests of the WPPSI/WISC test, including Block Design (BD), Matrix Reasoning (MR), and Picture Concept (PC), were administered to investigate relationships between the well-established tests and the outcomes with the TAG-Games measures. For ease of comparison, we calculated a composite score for each TAG-Game in a similar fashion to the method used in WPPSI and WISC. The WPPSI-IV was used for participants at the ages of four and five, with the WISC-V employed for children at the ages of six to eight. The three subtests from the WPPSI-IV/WISC-V were administered using the standardized protocol (i.e., instruction, prompts, time limits, and discontinuation rules) outlined in the manuals.

Two tables were set up to allow efficient administration of both TAG-Games and WPPSI/WISC tests simultaneously. TAG-Games were administered by an engineering graduate student trained for human subject studies and the WPPSI/WISC subtests were administered by an advanced psychology graduate student. Administration of TAG-Games in the current form of the technology only requires clicking icons on a graphical user interface to start and end each game using a mouse connected to an interfacing computer and replacing the blocks after completing each game item. Transition between the items can be easily automated, but this function was not incorporated at this early stage and we focused on evaluating sensor data accuracy and any potential technical problems. After completing each item, the blocks must be rearranged in randomized positions and orientations prior to displaying the next item. Unlike adult participants who could do this by themselves, the administrator relocated the blocks at each item completion for child participants. The order of administration between TAG-Games and WPPSI/WISC tests were altered. All participants completed both parts of the study within the same day. We note that the TAG-Games technology can support fully automated administration and data collection, while the current study involved a human administrator.

#### 2.2.3. Scoring methods

Scoring of TAG-Games considered correctness and time. For TAG-Game^A^ and TAG-Game^S^, terciles were employed for allocating different points based on the completion time for producing a correct answer. Completion times for all correct answers for each item were recorded and divided into three groups: correct answers made within 33% quantile time, correct answers made in between 33 and 67% quantile times and those took longer than 67% quantile time. Children often failed to produce correct assemblies in TAG-Game^A^-HF and TAG-Game^A^-AF. This was likely due to similarity in the appearance of the individual sides of a block. In these two games, for each item, up to two trials were allowed. For correct answers made in the first trial, 4–6 points were allocated depending on completion time. Correct answers made in the second trial after failing in the first trial received 1–3 points. For TAG-Game^M^, only correctness was considered. This game involves two items with the length from 1 to 6, and therefore, if a person successfully remembers items up to four-image sequences, the total score would be 20 (from 1+1+2+2+3+3+4+4 = 20). All incorrect answers received 0 point. For WPPSI-IV and WISC-V subtests, we used the scoring methods as outlined in the manual. The total available score of each subtest was 66 for BD, 26 for MR, and 48 for PC.

#### 2.2.4. Data analysis

TAG-Game scores, computed complexity values, and the scores from the three subtests of WPPSI-IV and WISC-V were the primary outcome measures used to examine the preliminary utility of TAG-Games for cognitive assessment. For each TAG-Game, the average raw score and scaled score were calculated to enable comparisons among the tests. Scaling was performed to convert a raw score to 0–100 scale. The data analysis strategy focused on establishing the preliminary psychometric integrity and utility of the measure in young children. As the first step, split-half reliability test was conducted using Spearman's correlations with α = 0.05. To evaluate the computational complexity measures defined in (1), we also investigated correlations between the complexity value computed for each item and the participant's performance based on time and correctness. For preliminary utility evaluation of TAG-Games for cognitive assessment capabilities, correlation analysis between the TAG-Games and WPPSI-IV/WISC-V were conducted.

## 3. Results

### 3.1. Results from TAG-games

As detailed in section 2.2.3, total available scores vary across different TAG-Games. Therefore, the raw scores obtained from the participants were transformed to a 0–100 scale for ease of comparison. Table 3 summarizes the results. The transformed scores for TAG-Game^A^ were 55.05, 59.44, and 67.08 for the three subtests, respectively. The two subtests in TAG-Game^S^ using the blocks with geometric shapes (GS) and emoticons (EM) resulted in similar average scaled scores, i.e., 45.97 and 48.22 for GS and EM, respectively. For the three TAG-Game^M^, the average scaled scores were 29.95, 21.19, and 23.57, respectively. As shown in the table, age correlations were found in the scores in TAG-Game^A^-GS. In the rest of the tests, we also found age correlations except for relatively poor performance in 8-year-olds.

**Table 3:** Average scores of TAG-Games for each age group.

**Age**	**TAG-Game^A^**			**TAG-Game^S^**		**TAG-Game^M^**		
**(No. subjects)**	**GS**	**HF**	**AF**	**GS**	**EM**	**CL**	**GS**	**EM**
4 (7)	15.3	22.5	25.5	16.8	8.7	8.2	11.8	8.3
5 (7)	26.7	25.3	27.5	15.2	5.8	9.2	10.2	7.5
6 (11)	33.3	19.6	21.3	13.6	8	9.3	11.3	8.7
7 (8)	37.5	26.5	28.9	22.5	12.9	10.8	16.4	14.1
8 (7)	46.3	15.3	20.1	15.9	8	6.8	13	10.6
Total Ave. (40)	33.03	21.40	24.15	16.55	8.68	12.58	8.90	9.90
Total Ave. Scaled	55.05	59.44	67.08	45.97	48.22	29.95	21.19	23.57

The number inside the parenthesis indicates the total available raw score for each game. The bottom row shows the total average scores transformed to 0–100 scale for ease of comparison.

For the three TAG-Game^A^, children showed a higher mean score using the blocks with human faces (HF) (average score = 59.44) and with animal faces (AF) (average score = 67.08) compared to GS (average score = 55.05). These data provide preliminary evidence in support of our hypothesis that typically developing children would better perform tasks using familiar images than that with unfamiliar shapes. Between AF and HF, the average score from AF was higher than that from HF. As shown in Figure 4, all human faces share similar geometry, size, and proportion, while the animal faces used in the test are clearly distinctive in color and shape, possibly making it easier for children to match the blocks correctly. In TAG-Game^S^, the scaled score from the test using EM (48.22) was higher than that using GS (45.97). Again, as shown in Figure 4, EM appear to be easier to process and identify the missing piece in the pattern possibly because it only involves two discriminating factors, i.e., color (black vs. yellow) and emotion (smiley vs. angry). On the other hand, six geometric shapes involve color (original vs. inverse) and shape (plane, strip, or triangle). The results from the three tests in TAG-Game^M^ were consistent with the previous findings. Children performed the best in memorizing sequences of CL blocks where color is the only variable. The scaled score in TAG-Game^M^-EM was slightly higher than that in TAG-Game^M^-GS, also implying that sequences of EM were slightly easier to memorize than that using GS.

### 3.2. Split-half reliability of TAG-games

For each TAG-Game, the items were divided into odd and even numbers and the scores were compared between the two. For TAG-Game^A^ with geometric shapes, a high correlation was found between the odd numbered items and even numbered items, i.e., *r*_(10)_ = 0.90 (*p* < 0.05). TAG-Game^A^-HF and -AF use similar types of items that are human and animal faces segmented into 4, and thus the difficulty across these items was assumed to be similar. For 6 items in each of TAG-Game^A^-HF/AF, split-half correlations were *r*_(3)_ = 0.64 (*p* < 0.05) and *r*_(3)_ = 0.57 (*p* < 0.05), respectively. For the two types of TAG-Game^S^, split-half reliability test showed that *r*_(6)_ = 0.76 (*p* < 0.05) for TAG-Game^S^ -GS and *r*_(3)_ = 0.68 (*p* < 0.05) for TAG-Game^S^-EM. There were three subtests in TAG-Game^M^, resulting in *r*_(3)_ = 0.70 (*p* < 0.05) for color blocks (CL),*r*_(3)_ = 0.67 (*p* < 0.05) for GS, and *r*_(3)_ = 0.62 (*p* < 0.05) for EM.

### 3.3. Validity of *C*^*play*^

The utility of the computational measures of play complexity for TAG-Games was evaluated by examining the correlation between play complexity and performance measures (e.g., completion time and correctness) at the item level. For TAG-Game^A^-GS, the mean time required for completing each item and correctness of the answer were considered to be two indices of performance and used to examine the impact that play complexity had on performance. As anticipated, $C_{A}^{play}$ was strongly correlated with the completion time [*r*_(20)_ = 0.99, *p* < 0.05] and negatively correlated with the correctness [*r*_(20)_ = −0.73, *p* < 0.05]. The *C*^*play*^ values across the items in TAG-Game^A^-HF/AF remain the same using the proposed formula because they all involve quadrants of face images ($C_{A}^{play}=22.92$). Therefore, those games were excluded from the analysis.

TAG-Game^S^-GS and EM employed the same performance indices, i.e., the mean time for completion and correctness, to correlate the performance with $C_{S}^{play}$. For TAG-Game^S^-GS, a strong correlation between $C_{s}^{play}$ and the average completion time was found [*r*_(12)_ = 0.76, *p* < 0.05] and a negative correlation between $C_{S}^{play}$ and correctness was found [*r*_(12)_ = −0.65, *p* < 0.05]. Similarly in TAG-Game^S^-EM, $C_{S}^{play}$ was strongly correlated with the average completion time [*r*_(6)_ = 0.84, *p* = 0.07] and negatively correlated with the correctness [*r*_(6)_ = −0.91, *p* < 0.05]. For the three subtests of TAG-Game^M^-CL, GS, and EM, only correctness was used in this evaluation. Strong negative correlations were found in all three TAG-Game^M^, resulting in *r*_(6)_ = −1.0(*p* < 0.05), *r*_(6)_ = −0.99(*p* < 0.05), and *r*_(6)_ = −1.0(*p* < 0.05) for CL, GS, and EM, respectively. Thus, the strong relationship between play complexity and our TAG-Games was found, implying the potential utility of *C*^*play*^ for personalized assessment.

### 3.4. Relationship between TAG-games and WPPSI/WISC

Table 4 shows the correlations among the TAG-Games and the subtests of WPPSI/WISC scores. Within the TAG-Games, strong correlations were found between the same types of TAG-Games, i.e., TAG-Game^A^ -GS was highly correlated with TAG-Game^A^-HF (*r* = 0.54, *p* < 0.05) and TAG-Game^A^-AF (*r* = 0.59, *p* < 0.05). Correlation between TAG-Game^A^-HF and TAG-Game^A^-AF was even higher (*r* = 0.65, *p* < 0.05). TAG-Game^S^-GS was highly correlated with TAG-Game^S^-EM (*r* = 0.62, *p* < 0.05). Three types of TAG-Game^M^ were also correlated with each other. We also found correlations across different types of TAG-Games. TAG-Game^A^-GS was correlated with TAG-Game^S^-GS (*r* = 0.67, *p* < 0.05), TAG-Game^S^-EM (*r* = 0.71, *p* < 0.05), and TAG-Game^M^-GS (*r* = 0.63, *p* < 0.05). TAG-Game^S^-GS showed relatively strong correlations with all three TAG-Game^M^.

**Table 4:** Correlation coefficients, *r*, among TAG-Games and WPPSI/WISC subtests.

	**TA-HF**	**TA-AF**	**TS-GS**	**TS-EM**	**TM-CL**	**TM-GS**	**TM-EM**	**BD**	**MR**	**PC**
TA-GS	0.54	0.59	0.67	0.71	0.47	0.63	0.42	0.49	0.40	0.26
	(< 0.05)	(< 0.05)	(< 0.05)	(< 0.05)	(< 0.05)	(< 0.05)	(< 0.05)	(< 0.05)	(< 0.05)	(0.11)
TA-HF	–	0.65	0.55	0.43	0.34	0.51	0.24	0.32	0.28	0.36
		(< 0.05)	(< 0.05)	(< 0.05)	(< 0.05)	(< 0.05)	(0.13)	(< 0.05)	(0.08)	(< 0.05)
TA-AF	–	–	0.58	0.41	0.39	0.45	0.26	0.46	0.39	0.24
			(< 0.05)	(< 0.05)	(< 0.05)	(< 0.05)	(0.11)	(< 0.05)	(< 0.05)	(0.14)
TS-GS	–	–	–	0.62	0.64	0.60	0.56	0.31	0.37	0.30
				(< 0.05)	(< 0.05)	(< 0.05)	(< 0.05)	(0.05)	(< 0.05)	(0.06)
TS-EM	–	–	–	–	0.45	0.50	0.48	0.14	0.35	0.20
					(< 0.05)	(< 0.05)	(< 0.05)	(0.39)	(< 0.05)	(0.21)
TM-CL	–	–	–	–	–	0.64	0.69	0.12	0.19	0.32
						(< 0.05)	(< 0.05)	(0.46)	(0.25)	(< 0.05)
TM-GS	–	–	–	–	–	–	0.54	0.26	0.26	0.23
							(< 0.05)	(0.10)	(0.11)	(0.15)
TM-EM	–	–	–	–	–	–	–	0.24	0.24	0.17
								(0.13)	(0.14)	(0.30)
BD	–	–	–	–	–	–	–	–	0.39	0.33
									(< 0.05)	(< 0.05)
MR	–	–	–	–	–	–	–	–	–	0.34
										(< 0.05)

*The values inside the parenthesis indicate the significance, p. For short handwriting, TA, TAG-Game^A^, TS, TAG-Game^S^, TM, TAG-Game^M^*.

First focusing on the raw scores in WPPSI/WISC, BDR was correlated with all three types of TAG-Game^A^ (GS: *r* = 0.49, HF: *r* = 0.32, AF:*r* = 0.46; *p* < 0.05). BD was also correlated with TAG-Game^S^-GS (*r* = 0.31, *p* = 0.05), but not correlated with TAG-Game^S^-EM. MR showed correlations with TAG-Game^A^-GS (*r* = 0.40, *p* < 0.05), TAG-Game^A^-AF (*r* = 0.39, *p* < 0.05), and the two types of TAG-Game^S^ (GS: *r* = 0.37, EM: *r* = 0.36; *p* < 0.05). PC was correlated with TAG-Game^A^-HF (*r* = 0.36, *p* < 0.05) and TAG-Game^M^-CL (*r* = 0.32, *p* < 0.05).

While some correlations between TAG-Game scores and raw scores from the three subtests of BD, MR, and PC were observed, no significant correlations were found between the TAG-Games scores and the standardized BD, MR, or PC scores as expected. This may be due to the fact that the WPPSI/WISC are standardized measures and the scaled scores take into account the age differences, while TAG-Games has not been standardized. This is also reflected in the age correlation data shown in Table 5. TAG-Game scores showed higher age correlations than the BD, MR, and PC raw scores, implying that TAG-Games may better capture developmental differences than the Wechsler's subtests.

**Table 5:** Age correlations for the TAG-Game scores, the raw WPPSI/WISC scores (i.e., Block Design raw score (BDR), Matrix Reasoning raw score (MRR), and Picture Concept raw score (PCR), and the standardized WPPSI/WISC scores (i.e., Block Design standardized score (BDS), Matrix Reasoning standardized score (MRS), and Picture Concept standardized score (PCS).

**TA-GS**	**TA-HF**	**TA-AF**	**TS-GS**	**TS-EM**	**TM-CL**	**TM-GS**	**TM-EM**	**BDR**	**MRR**	**PCR**	**BDS**	**MRS**	**PCS**
0.74	0.70	0.70	0.63	0.63	0.48	0.60	0.39	0.39	0.41	0.34	0.02	−0.17	0.07
(< 0.05)	(< 0.05)	(< 0.05)	(< 0.05)	(< 0.05)	(< 0.05)	(< 0.05)	(< 0.05)	(< 0.05)	(< 0.05)	(< 0.05)	(0.88)	(0.30)	(0.68)

## 4. Discussion

### 4.1. Conclusion

Forty children aged between 4 and 8 participated in our preliminary evaluation. In TAG-Game^A^ (block assembly tasks), the average scaled score in the test using the geometric shapes was lower than the tests using the images of human or animal faces. Between the tests using human faces and animal faces, the average scaled score in the animal face test was significantly higher than the test with human faces. It is possibly because the animal faces used in the items were more distinctive than human faces in color and geometry. Children showed better performance in TAG-Game^S^ (shape matching tasks) involving the emoticon images than TAG-Game^S^ with geometric shapes as well. In TAG-Game^M^ (sequence memory tasks), the results were consistent with the other two types of TAG-Games, showing that the children could better memorize sequences of simpler or familiar images than those involving unfamiliar geometric shapes. Internal consistency was examined by the split-half reliability test for all TAG-Games. A significant correlation was found in each test, implying internal consistency in the test design.

The proposed computational measures of *C*^*play*^ were strongly correlated to task performance measured by completion time and correctness, implying their potential roles for dynamically adjusting test difficulty to address individual or group differences. In order for cognitive assessment to produce sensitive assays, it is important for the test to involve sensitive items appropriate for each group or individual. For the target population presented in this paper, we manually adjusted test items from the original version used for young adults (age: 19–30). If fully validated, *C*^*play*^ can serve as a useful tool to automatically generate easier or harder items based on age and cognitive status. The relationship between TAG-Games and WPPSI/WISC subtests were also analyzed by examining correlations among the test scores. While some weak to moderate correlations were found between the TAG-Games and WPPSI/WISC subtests, further evaluations are needed to produce valid comparisons between the two sets of tests.

### 4.2. Limitation and potential

The small sample size for each age group does not allow for a definite statement regarding the reliability and validity of this measure. The current prototype system, which is all handmade and lab fabricated, still requires an administrator to check if the blocks function properly time to time. The battery power must be extended. The current blocks need to be recharged after 2 h of continuous use. We encountered occasional technical malfunctions, mostly related to infrared (IR) sensors installed on the block surfaces which were connected to the main circuit with a removable socket for easy replacement. Once fully established and professionally manufactured, technical errors will be nearly zero with enhanced battery performance with optimized circuit design for reduced power consumption.

The defined *C*^*play*^ formulas for the task complexity associated with each type of TAG-Games showed significant positive correlations with completion time (i.e., the higher *C*^*play*^ the longer it takes to complete) and significant negative correlations with accuracy. The evaluation for $C_{A}^{play}$ was only performed on the TAG-Game^A^ with geometric shapes results, because its items have varying $C_{A}^{play}$ values while TAG-Game^A^ with the human or animal face images have the same $C_{A}^{play}\left(=22.92\right)$ value across all items. This value is the maximum available value for $C_{A}^{play}$ for a 4-block item. This is because the task requires the player to identify one of the six images on each block and place it in an exact position and orientation without allowing permutation. However, children's performance in the items with the same complexity values resulted in significant differences in task performance depending on the types of block images used, i.e., geometric shapes, colors, emoticons, human faces, and animal faces. This is because the proposed complexity measure was defined based on discrete entropy changes by assembling the blocks, without taking account of the possible “familiarity” and “distinctive” factors. To address this limitation in the current definitions of *C*^*play*^, different image processing approaches could be used to quantify color and shape variation as well as familiarity factors.

With these limitations in mind, the TAG-Games system is uniquely positioned as an automated assessment tool for cognitive skills through tangible manipulation of the blocks. The games do not require the use of language, potentially reducing cultural bias. TAG-Games have the potential for addressing limitations in the traditional assessment methods by (1) reducing cost by automating the process and therefore reducing or eliminating clinician/administrator time, (2) improving the quantity and quality of the measurable data, (3) enabling objective assessment, and (4) enabling wireless, remote administration for hard-to-reach areas. Once the proposed computational measures are fully validated, TAG-Games can be fully customized and tailored for each individual or group, potentially increasing sensitivity in assays. While the presented results suggest the potential of TAG-Games for cognitive assessment in children, the data here must be interpreted with caution.

### 4.3. Future work

Our future work will focus on further validation of this new technology-based cognitive assessment for children by (1) continuing human subject evaluation to achieve a significant statistical power in the data, (2) improving the technology to be more user friendly (e.g., longer battery life, easy-to-use charging station, and enhanced graphics in the user interface), and (3) randomizing the order of the games and employed tests. Our team is highly interested in investigating the potential utility of this technology for in-home cognitive assessment for children who require continuous monitoring of their cognitive skills, such as those with attention deficits and hyperactivity disorder (ADHD), learning disabilities, autism spectrum disorder (ASD), and other cognitive or behavioral problems. To do so, our second phase of human subject study will involve two groups of children, healthy and cognitively delayed, and examine group differences and how TAG-Games can provide detailed information on each individuals cognitive performance and behavior changes over time. Further technical improvements would be necessary for this study to be conducted at each participant's home.

## Author contributions

KL: As the principal investigator of this project, she developed the technology, including SIG-Blocks and associated software algorithms. She also developed computational play complexity measures. DJ: As the graduate research assistant, he built the technology under supervision of KL. RS, LH, SG: As graduate research assistants of Psychological Sciences, they administered the Wechsler's tests on young children under supervision of ES. ES: As the co-investigator of this project, she collaborated on game design, designed the study protocols, and led the human subject study.

### Conflict of interest statement

The authors declare that the research was conducted in the absence of any commercial or financial relationships that could be construed as a potential conflict of interest. The reviewer BB and handling Editor declared their shared affiliation.

## References

[1] Flavell JH Cognitive development: past, present, and future. Dev Psychol. (1992) 28:998 10.1037/0012-1649.28.6.998

[2] Wertsh JV Tulviste P Apprenticeship in thinking: cognitive development in social context. Science (1990) 249:684–6. 10.1126/science.249.4969.684

[3] Robins DL Fein D Barton ML Green JA. The Modified Checklist for Autism in Toddlers: an initial study investigating the early detection of autism and pervasive developmental disorders. J Autism Dev Disord. (2001) 31:131–44. 10.1023/A:1010738829569 11450812

[4] Chakrabarti S Fombonne E. Pervasive developmental disorders in preschool children: confirmation of high prevalence. Am J Psychiatry (2005) 162:1133–41. 10.1176/appi.ajp.162.6.1133 15930062

[5] Wechsler D The Measurement of Adult Intelligence Scale. Baltimore, MD: Williams and Wilkins (1944).

[6] Littell WM The wechsler intelligence scale for children: review of a decade of research. Psychol Bull. (1960) 57:132 10.1037/h0044513 14417533

[7] Strauss E Sherman EM Spreen O A Compendium of Neuropsychological Tests: Administration, Norms, and Commentary. New York, NY: Oxford University Press (2006).

[8] Raven JC Court JH Raven J Manual for Raven's progressive matrices and vocabulary scales. London: Lewis (1988).

[9] Raven J. The Raven's progressive matrices: change and stability over culture and time. Cogn Psychol. (2000) 41:1–48. 10.1006/cogp.1999.0735 10945921

[10] Diaz RM Chapter 2: thought and two languages: the impact of bilingualism on cognitive development. Rev Res Educ. (1983) 10:23–54. 10.3102/0091732X010001023

[11] Bors DA Stokes TL Raven's advanced progressive matrices: norms for first-year university students and the development of a short form. Educ Psychol Meas. (1998) 58:382–98. 10.1177/0013164498058003002

[12] Siu AL Bibbins-Domingo K Grossman DC Baumann LC Davidson KW Ebell M. (2016). Screening for autism spectrum disorder in young children: US Preventive Services Task Force recommendation statement. JAMA 315:691–6. 10.1001/jama.2016.0018 26881372

[13] Goldin-Meadow S Levine SC Hedges LV Huttenlocher J Raudenbush SW Small SL. New evidence about language and cognitive development based on a longitudinal study: hypotheses for intervention. Am. Psychol. (2014) 69:588. 10.1037/a0036886 24911049 PMC4159405

[14] Smits-Engelsman B Wilson P Westenberg Y Duysens J. Fine motor deficiencies in children with developmental coordination disorder and learning disabilities: an underlying open-loop control deficit. Hum Movem Sci. (2003) 22:495–513. 10.1016/j.humov.2003.09.006 14624830

[15] Cameron CE Cottone EA Murrah WM Grissmer DW How are motor skills linked to children's school performance and academic achievement? Child Dev Perspect. (2016) 10:93–8. 10.1111/cdep.12168

[16] Raz-Silbiger S Lifshitz N Katz N Steinhart S Cermak SA Weintraub N. Relationship between motor skills, participation in leisure activities and quality of life of children with developmental coordination disorder: temporal aspects. Res Dev Disabil. (2015) 38:171–80. 10.1016/j.ridd.2014.12.012 25589477

[17] Wray C Norbury CF Alcock K. Gestural abilities of children with specific language impairment. Int J Lang Commun Disord. (2016) 51:174–82. 10.1111/1460-6984.12196 26303884

[18] Wechsler D Manual for the Wechsler Adult Intelligence Scale (1955).

[19] Watkins MW Smith LG. Long-term stability of the Wechsler Intelligence Scale for Children–Fourth Edition. Psychol Assess. (2013) 25:477. 10.1037/a0031653 23397927

[20] Joy S Fein D Kaplan E Freedman M. Speed and memory in WAIS-R-NI digit symbol performance among healthy older adults. J Int Neuropsychol Soc. (2000) 6:770–80. 10.1017/S1355617700677044 11105467

[21] Kulp MT Relationship between visual motor integration skill and academic performance in kindergarten through third grade. Optomet Vis Sci. (1999) 76:159–63. 10.1097/00006324-199903000-00015 10213445

[22] Glascoe FP Foster EM Wolraich ML. An economic analysis of developmental detection methods. Pediatrics (1997) 99:830–7. 10.1542/peds.99.6.830 9164778

[23] Lipkin PH Macias MM Hyman SL Coury DL O'Connor KG Identification of children < 36 months at risk for developmental delay/autism: results of national survey of pediatricians. J Dev Behav Pediatr. (2017) 3:S4–5.

[24] Committee on Children with Disabilities (2001). Developmental surveillance and screening of infants and young children. Pediatrics 108:192–5. 10.1542/peds.108.1.192 11433077

[25] Short EJ Noeder M Gorovoy S Manos MJ Lewis B The importance of play in both the assessment and treatment of young children. In Russ S. Niec L., editors. An Evidence-Based Approach to Play in Intervention and Prevention: Integrating Developmental and Clinical Science. New York, NY: Guilford (2011).

[26] Vrana SR Vrana DT Can a computer administer a wechsler intelligence test? Profes Psychol Res Pract. (2017) 48:191 10.1037/pro0000128

[27] Pearson (2016). Welcome to Q-interactive. Available online at: http://www.helloq.com/home.html

[28] Taur S Karande S Saxena AA Gogtay NJ Thatte UM. Use of computerized tests to evaluate psychomotor performance in children with specific learning disabilities in comparison to normal children. Ind J Med Res. (2014) 140:644. 25579146 PMC4311318

[29] Lumsden J Edwards EA Lawrence NS Coyle D Munafò MR. Gamification of cognitive assessment and cognitive training: a systematic review of applications and efficacy. JMIR Ser Games (2016) 4:e11. 10.2196/games.5888 27421244 PMC4967181

[30] Luciana M. Practitioner review: computerized assessment of neuropsychological function in children: clinical and research applications of the Cambridge Neuropsychological Testing Automated Battery (CANTAB). J Child Psychol Psychiatry (2003) 44:649–63. 10.1111/1469-7610.00152 12831110

[31] Jung J Kim L Park S Kwon GH E-CORE (Embodied COgnitive REhabilitation): a cognitive rehabilitation system using tangible tabletop interface. In: Converging Clinical and Engineering Research on Neurorehabilitation. Berlin; Heidelberg: Springer (2013). p. 893–7.

[32] Lee K Jeong D Schindler RC Short EJ SIG-Blocks: tangible game technology for automated cognitive assessment. Comput Hum Behav. (2016) 65:163–75. 10.1016/j.chb.2016.08.023

[33] Jeong D Lee K iSIG-Blocks: interactive creation blocks for tangible geometric games. IEEE Trans Consum Electr. (2015). 61:420–8. 10.1109/TCE.2015.7389795

[34] Jeong D Endri K Lee K TaG-games: tangible geometric games for assessing cognitive problem-solving skills and fine motor proficiency. In: IEEE Conference on Multisensor Fusion and Integration for Intelligent Systems (MFI). Salt Lake, UT: IEEE (2010). p. 32–37. 10.1109/MFI.2010.5604479

